# Columnar Connectivity and Laminar Processing in Cat Primary Auditory Cortex

**DOI:** 10.1371/journal.pone.0009521

**Published:** 2010-03-03

**Authors:** Craig A. Atencio, Christoph E. Schreiner

**Affiliations:** 1 Berkeley Bioengineering Graduate Group, University of California, Berkeley and University of California San Francisco, San Francisco, California, United States of America; 2 W.M. Keck Foundation Center for Integrative Neuroscience, San Francisco, California, United States of America; 3 Coleman Memorial Laboratory, Department of Otolaryngology-Head and Neck Surgery, University of California San Francisco, San Francisco, California, United States of America; Ludwig Maximilians University Munich, Germany

## Abstract

**Background:**

Radial intra- and interlaminar connections form a basic microcircuit in primary auditory cortex (AI) that extracts acoustic information and distributes it to cortical and subcortical networks. Though the structure of this microcircuit is known, we do not know how the functional connectivity between layers relates to laminar processing.

**Methodology/Principal Findings:**

We studied the relationships between functional connectivity and receptive field properties in this columnar microcircuit by simultaneously recording from single neurons in cat AI in response to broadband dynamic moving ripple stimuli. We used spectrotemporal receptive fields (STRFs) to estimate the relationship between receptive field parameters and the functional connectivity between pairs of neurons. Interlaminar connectivity obtained through cross-covariance analysis reflected a consistent pattern of information flow from thalamic input layers to cortical output layers. Connection strength and STRF similarity were greatest for intralaminar neuron pairs and in supragranular layers and weaker for interlaminar projections. Interlaminar connection strength co-varied with several STRF parameters: feature selectivity, phase locking to the stimulus envelope, best temporal modulation frequency, and best spectral modulation frequency. Connectivity properties and receptive field relationships differed for vertical and horizontal connections.

**Conclusions/Significance:**

Thus, the mode of local processing in supragranular layers differs from that in infragranular layers. Therefore, specific connectivity patterns in the auditory cortex shape the flow of information and constrain how spectrotemporal processing transformations progress in the canonical columnar auditory microcircuit.

## Introduction

The thalamocortical synapse sets the stage for the cortical delineation and integration of auditory information. The sequence of processing and the flow of information are governed by stereotypical and precise connections between cortical laminae. Layer 4 neurons respond with the shortest latency, followed by those in layers 2/3 and 5 [1]. Although minimal latencies have been observed in the deep layers of auditory cortex for rodent and guinea pig [2], [3], [4], the predominant input from the thalamus to cortex nevertheless arrives in layer 4 and deep layer 3 [5], [6]. The outputs of layers contribute to functional circuits via vertical and horizontal cortical connections, allowing different positions in the cortical network to be influenced by recurrent activity [7], [8], [9], [10] [11] [12].

Four approaches have been predominately used to determine how neurons are functionally connected and how information is distributed in the AI microcircuit. Antidromic stimulation and focal tracer injection studies have delineated the general scheme of columnar laminar connectivity [7], [8], [13], [14]. Both response latency and local field potentials have been used to map the laminar flow of information [1], [2], [3], [15]. Response latency relates to initial timing, and electrical stimulation and tracer studies provide anatomical confirmation and frameworks. These approaches do not, however, reveal the responses of neurons to complex stimuli or synchronization within cortical columns and, therefore, cannot disclose how functional connectivity relates to cortical processing principles and emerging receptive field properties.

In the primary visual cortex (VI), response synchrony between layers has been more extensively characterized. Anatomical studies delineated the strength of connections, the probability of finding connectivity, and the local schemes that help to define the VI microcircuit [16], [17], [18]. These approaches have been complemented by in-vivo studies, which focused on functional aspects of connectivity, and, for example, revealed the consequences of direct input from layer 4 simple cells to layer 2/3 complex cells [19], which represents a major cortical processing transformation.

In somatosensory cortex, interlaminar connections were found to be precise between supragranular and infragranular cells [20], [21], [22], [23]. These studies revealed the strongest synchronization in layer 5, and the weakest in layer 4. Temporal interactions were strongest between neurons in the same barrel, though how these interactions correspond to receptive field transformations remains unresolved.

In auditory cortex, functional connectivity studies have focused on properties of horizontal projections [24], [25], [26], [27], [28], [29]. The strength of horizontal connections was found to vary with the distance between neurons [25], [30]. By using simple sounds, the strength of connections between neurons at different locations within AI could be predicted [29]. However, little work has addressed the functional connectivity of neurons across AI layers. An in-vitro study in rat AI has revealed a connectivity scheme similar to other sensory cortices, though matching in-vivo studies of functional response characteristics are still lacking [31]. Since we do not have detailed knowledge of the in-vivo functional connectivity between layers, and their relation to receptive field behavior, we have an incomplete framework for understanding complex auditory information processing in auditory cortical microcircuits.

We attempted to illuminate the link between functionally defined connectivity and processing characteristics in the columnar circuit by simultaneously recording from multiple neurons in different AI laminae. We stimulated with a complex sound and quantified the functional connectivity between neuron pairs, which we then related to spectrotemporal receptive field (STRF) parameters. We calculated pair-wise correlations between recorded neurons and examined how functional connectivity varied within and between laminae, how it varied with synaptic distance, how receptive field parameters varied with connection strength, and how connectivity between layers related to receptive field similarity. These results provide a quantitative estimate of the relationships between local connectivity, the columnar representation of receptive field properties, and information flow in the auditory cortical microcircuit.

## Methods

### Electrophysiology

All experimental procedures were approved by the University of California, San Francisco Committee for Animal Research. The experimental procedures used in this study have been previously described [32]. Briefly, young adult cats (N = 10) were given an initial dose of ketamine (22 mg/kg) and acepromazine (0.11 mg/kg), and then anesthetized with pentobarbital sodium (Nembutal, 15–30 mg/kg) during the surgical procedure. The animal's temperature was maintained with a thermostatic heating pad. Bupivicaine was applied to incisions and pressure points. Surgery consisted of a tracheotomy, reflection of the soft tissues of the scalp, craniotomy over AI, and durotomy. After surgery, to maintain an areflexive state, the animal received a continuous infusion of ketamine/diazepam (2–5 mg/kg/h ketamine, 0.2–0.5 mg/kg/h diazepam in lactated Ringer solution).

With the animal inside a sound-shielded anechoic chamber (IAC, Bronx, NY), stimuli were delivered via a closed speaker system to the ear contralateral to the exposed cortex (diaphragms from Stax, Japan). Extracellular recordings were made using multi-channel silicon recording probes, which were provided by the University of Michigan Center for Neural Communication Technology [33]. The probes contained sixteen linearly spaced recording channels, with each channel separated by 150 µm. We only used probes with channel impedances between 2 and 3 MΩ, since these impedances allowed us to resolve single units. Probes were carefully positioned orthogonally to the cortical surface and lowered to depths between 2300 and 2400 µm using a microdrive (David Kopf Instruments, Tujunga, CA).

Neural traces were bandpass filtered between 0.6 and 6 kHz and were recorded to disk with a Neuralynx Cheetah A/D system at sampling rates between 18 kHz and 27 kHz. After each experiment the traces were sorted off-line with a Bayesian spike sorting algorithm [34]. Only events in the traces that exceeded the DC baseline by 5 RMS noise levels were used in the spike sorting procedure. Most channels of the probe yielded 1–2 well-isolated single units. All recording locations were in AI, as verified through initial multi-unit mapping and determined by the layout of the tonotopic gradient and bandwidth modules on the crest of the ectosylvian gyrus [35].

Penetrations with the linear recording array were orthogonal to the cortical surface and spanned all cortical layers. We operationally refer to this recording approach as ‘columnar’: the activity of recorded neurons represents processes that span the full vertical thickness of the cortical laminae, but may include more interactions than represented by the extent of anatomical microcolumns, and less than the extent of functional modules [36]. The positions of recorded neurons relative to cytoarchitectonic laminae were estimated based on a combination of depth estimate of the recording electrode relative to the cortical surface, first spike latency profile and, if available, current-source-density (CSD) profiles [37], [38]. The depth ranges were used as a predominant criterion after verification with latency and CSD measures in several penetrations, and were in accord with established AI laminar boundaries [8], [13], [14], [39]. Laminar assignment differences affected by local changes in cortical thickness were minimized since depth readings were aligned with a functional estimate of the granular layer position. To further reduce measurement noise due to electrode placement and local functional or anatomical variations, we defined laminar boundaries to be: layer 2 (200–375 µm); layer 3 (450–725 µm); layer 4 (800–1100 µm); layer 5 (1175–1500 µm); and layer 6 (1575–2000 µm). Neurons that fell into the 75 µm intervals between these layer ranges were considered to be of ambiguous designation and were not considered for laminar group analysis.

### Stimulus

Neurons were probed with pure tones, then with one or two presentations of a 15 or 20 minute dynamic moving ripple stimulus. The level and frequency of each pure tone was chosen randomly from 15 different levels (5 dB spacing) and 45 different frequencies. Each pure tone was presented 5 times at a given level and frequency. The ripple stimulus was a temporally varying broadband sound (0.5–20 or 40 kHz) composed of approximately 50 sinusoidal carriers per octave, each with randomized phase [40]. The magnitude of a carrier at any time was modulated by the spectrotemporal envelope. The envelope was defined by a spectral and a temporal modulation parameter. Spectral modulation rate is defined by the number of spectral peaks per octave. Temporal modulation rate is defined as the number of peaks per second. Both the spectral and temporal modulation parameters varied independently and randomly. Spectral modulation rate varied between 0 and 4 cycles per octave. The temporal modulation rate varied between −40 Hz (upward sweep) and 40 Hz (downward sweep). Both parameters were statistically independent and unbiased within these ranges. Maximum modulation depth of the spectrotemporal envelope was 40 dB. The mean intensity was set between 50–70 dB SPL, which was approximately 30–50 dB above the average pure-tone threshold within a given penetration.

### Analysis

Data analysis was carried out in MATLAB (Mathworks, Natick, MA). For each neuron, frequency response areas (FRAs) were computed from the pure tone responses, while the spike-triggered average of the spectrotemporal envelope immediately preceding a spike was used to derive the spectrotemporal receptive field (STRF) [40], [41], [42], [43], [44]. STRFs were thresholded so that only significant features (p<0.01) were included in the analysis [40].

Modulation properties were derived by computing the two-dimensional Fourier transform (FT) of each STRF. The FT is a function of temporal (cycles/s) and spectral modulation rate (cycles/octave). The magnitude of this function was folded along the vertical midline (temporal modulation frequency  = 0) to obtain the Ripple Transfer Function (RTF). Since the FT is sensitive to periodicities in the STRF, the RTF reflects the relationship of excitatory (ON) and suppressive (OFF) STRF subfields. RTFs were used to obtain modulation transfer functions (MTFs). By summing the RTF along the spectral modulation axis, we obtained the temporal modulation transfer function (tMTF). We obtained the spectral modulation transfer function (sMTF) by summing along the temporal modulation axis. MTFs were classified as bandpass if, after identifying the peak in the MTF, values at lower and higher modulation rates decreased by at least 3 dB. If there was no such decrease the MTF was classified as lowpass. Highpass MTFs were not encountered. Best modulation rate for bandpass MTFs was the rate corresponding to the peak value in the MTF, while for lowpass MTFs it was the mean between the 0 modulation frequency value and the 3 dB high side cutoff.

To analyze the temporal relationship between discharges of neurons in cortical layers, we followed standard cross-covariance procedures to estimate both the strength of temporal interactions between neurons and the error bounds on our parameter estimates [45], [46], [47]. First, spike trains were obtained by binning the spike times for each neuron with 1 ms resolution. For a single spike train 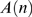, *n* is the bin number and 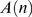 is either 1 (spike) or 0 (no spike). For two spike trains 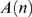 and 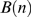, the mean intensities, *P_A_* and *P_B_*, for a sample of duration *D* bins, are estimated as 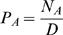 and 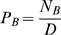, where *N_A_* and *N_B_* are the total number of spikes in trains *A* and *B*, respectively. For the spike trains in this study the stimulus duration was either 15 or 20 minutes, giving *D* = 900,000 or 1,200,000 bins.

The cross-correlation function between spike trains 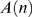 and 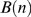 is then estimated as
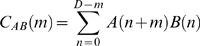



From 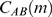 an unbiased estimate of the second order cross-product density, 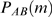, is
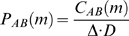
where 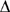 is the bin size of the spike train, in milliseconds. The cross-covariance function, 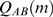, is then defined as
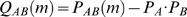



Thus, the cross-covariance function is a scaled version of the cross-correlation function, with the mean background activity removed. Cross-covariance values that are approximately zero represent chance coincidences between the two spike trains. Deflections from zero represents how the activity of one neuron influences the firing of the other neuron. Note that 
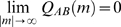
. The cross-covariance function 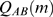 has an asymptotic distribution from which its variance can be estimated [47]. Under the assumption of independent Poisson spike trains, the variance of 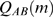 may be approximated as
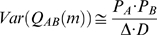



Thus, for two spike trains, with a 1 ms bin size, upper and lower 99% confidence limits (CL) for 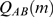 can be set at
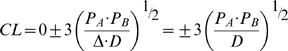



Only cross-covariance functions with two consecutive bins satisfying the 99% confidence limits were analyzed in this study.

Following earlier work, we calculated the correlation coefficient for each pair of neurons [25], [30]. The correlation coefficient is a measure of the peak connection strength and is defined by
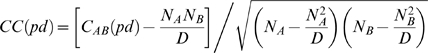
where *pd* is the delay at which the peak value in the cross-correlation function, *C_AB_*, occurs, with the other variables as previously defined.

For each of pair of neurons we also computed the Similarity Index (SI) between the STRFs [40]. The SI ranges between +1 and −1 and is a measure of the spectrotemporal correlation between the two receptive fields. For two STRFs, represented in matrix form as x*(i,j)* and y*(i,j)*, the SI is defined as
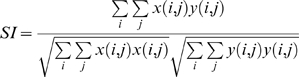
where *i* and *j* range over the number of rows and columns in the STRF.

To determine the stimulus selectivity of each neuron we calculated a feature selectivity index (FSI) for each neuron [40], [48]. For each action potential emitted by the neuron, the ripple envelope that preceded the spike was captured and correlated with the STRF of the neuron to obtain the similarity index. A similarity index value was calculated for each action potential, forming a SI probability distribution, 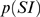. After calculating 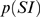 for stimulus segments that triggered an action potential, we then created a spike train of 10,000 random spikes [32], [48]. From the random spikes we again calculated *SI*s, and formed a probability distribution, 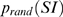. For each SI probability distribution, the cumulative density function was then calculated according to
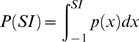



For the random spike train 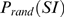 will contain a sharp upward transition near SI = 0, while for a neuron that is selective for only one stimulus feature 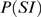 will contain a sharp upward transition near SI = 1. To quantify the difference between the random and recorded spike trains we then computed the areas under each cumulative density function via
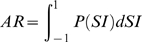
from which we then calculated the FSI as
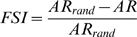



FSI values vary between 0 and 1, where 0 corresponds to similar distributions for 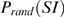 and 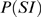 and thus to a neuron that responds indiscriminately to stimulus segments. When 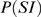 represents a distribution for a very stimulus selective neuron, AR will be near 0 and the FSI value will be approximately 1, corresponding to a neuron that is selective for relatively few stimulus features.

The separability of the STRF was determined by performing singular value decomposition [49]. The separability was defined as 
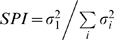
, where 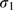 is the largest singular value. The SPI, which ranges between 0 and 1, describes how well the STRF may be described by a product of two 1D functions: one a function of time and the other a function of frequency, with values near 1 corresponding to an STRF for which time and frequency may be dissociated.

Using previously described methodologies, we computed a phase locking index (PLI) for each neuron using the relation 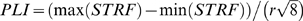, where max(STRF) and min(STRF) are the maximum and minimum values in the STRF, and *r* is the average firing rate [40]. The PLI ranges from 0 (not phase locked) to 1 (precisely phase locked) to the stimulus envelope.

## Results

In this study, we characterized the functional connectivity of AI neurons across multiple laminae, and related it to processing properties reflected in spectrotemporal receptive fields. We made 76 orthogonal penetrations in AI using multi-channel probes and simultaneously recorded the responses of 1100 neurons at different positions. After analysis, we obtained 8364 pairs of functionally connected neurons. The majority of the penetrations were obtained on the crest of the ectosylvian sulcus, in the central, more sharply tuned region of AI with characteristic frequencies between 8 and 23 kHz. In each penetration, we probed the responses of neurons by challenging them with a dynamic moving ripple stimulus. We usually obtained 1–2 single units per channel. From the isolated action potentials we constructed STRFs (see [50] for an example of spike shapes of multiple single units recorded from the same electrode channel).

### Example STRFs

**Figure 1:**
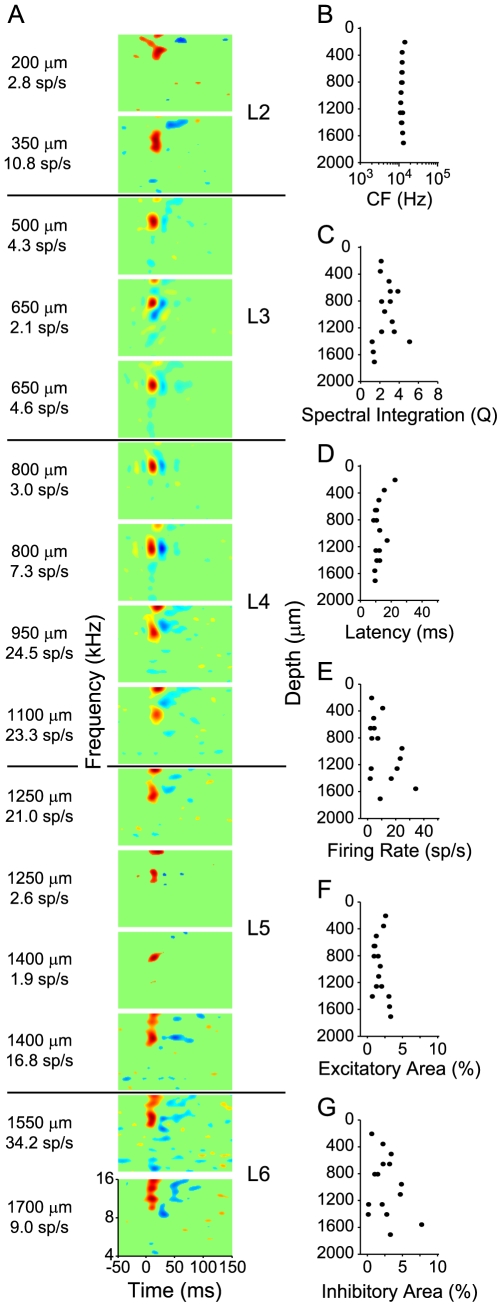
Example multi-channel recording, with spectrotemporal receptive fields (STRFs) and response parameters. (A) STRFs from simultaneously recorded columnar neurons in AI. Each row represents a single neuron. The cortical depth and firing rate of each neuron are indicated to the left of the STRFs. STRFs with the same depth values indicate that multiple neurons were recorded from the same electrode channel. (B) Characteristic frequency (CF) from the excitatory subfield of the STRFs. (C) Spectral integration, or quality factor (Q), of the excitatory subfield of the STRFs. (D) Peak excitatory latency in the STRFs. (E) Firing rate over the ripple stimulus duration. (F) STRF excitatory area percentage, or proportion of pixels, in the STRFs that were excitatory. (G) STRF inhibitory area percentage. Excitatory and inhibitory area percentages were determined by dividing the number of excitatory or inhibitory pixels by the total number of STRF pixels.

In the majority of penetrations, multiple single units could be isolated. One exemplary penetration is shown in Figure 1, which contains STRFs of 15 single units reconstructed at different positions across the vertical axis of the cortical sheet. The depth of each neuron from the cortical surface is indicated to the left of each STRF. In many cases, two neurons were recorded from the same electrode channel. The STRF depth profile reveals that for the main excitatory STRF subfield (red), latencies are shortest in layers 3b/4 and 6, which receive thalamic input (Fig. 1D).

The structure of the STRFs changed with depth, although STRFs within several hundred microns of each other were often quite similar. In infragranular layers, STRF structure was most varied, especially with regard to the structure of inhibitory subfields (blue) (Fig. 1A). These layer-dependent changes were a commonly observed feature in our data set.

For each penetration, we derived multiple parameters from the STRFs, and plotted them versus depth (Fig. 1 B–G). For the example penetration, characteristic frequency (CF) was relatively constant across position (Fig. 1B). Spectral integration in this penetration, as determined from the quality factor (Q  =  CF/Excitatory Bandwidth), broadened from layer 3 to layer 5 (Fig. 1C), indicating broader tuning in infragranular layers. The quality factor may change with depth due to a decrease in sideband inhibition as depth increases [51], [52]. The latency of the peak STRF response was consistent with previous laminar definitions (Fig. 1D). Minimum values occurred between 600–1100 µm, consistent with thalamic projection patterns [7]. However, latencies in infragranular layers could be quite short, similar to granular layer responses. Evoked firing rate also varied with depth, and was highest in deep granular and infragranular layers (>1100 µm; Fig. 1E). Last, we examined how much area in the STRF was occupied by the excitatory or the inhibitory subfields. The percent area was determined by first calculating the number of pixels in the STRF that corresponded to either the excitatory or inhibitory subfields. We then divided that number by the total number of pixels in the STRF to derive a normalized estimate. The excitatory area was contained within a restricted range of percentages, with the higher values in layer 6 reflecting broader excitatory tuning (Fig. 1F). In contrast, the percent area for the inhibitory subfields was variable, and did not follow a consistent depth profile (Fig. 1G).

### STRFs and Cross-Covariance Functions

**Figure 2:**
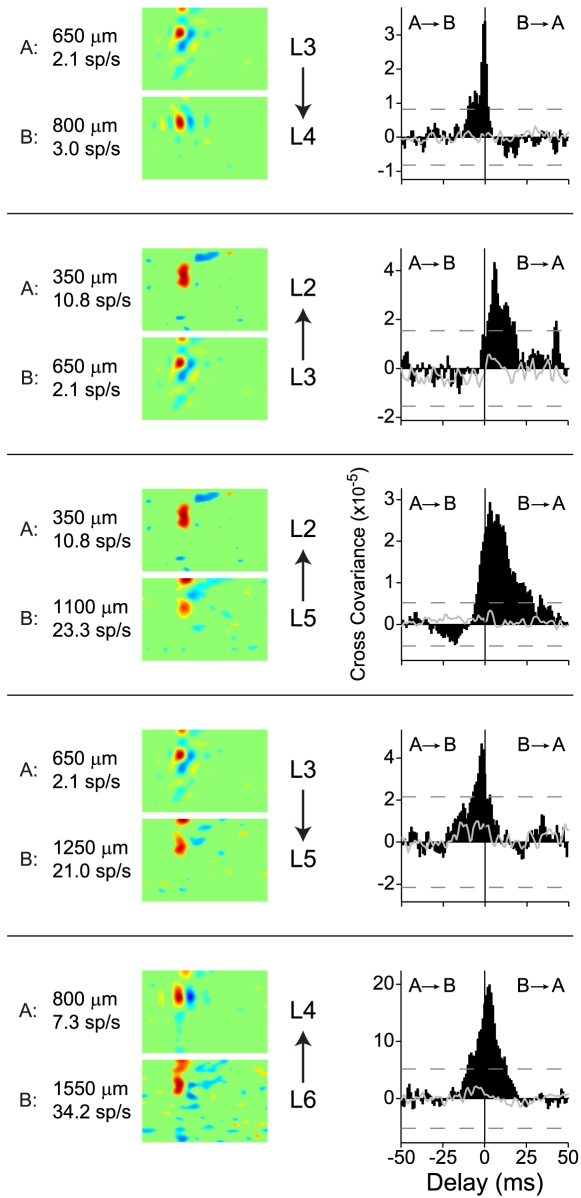
Example STRFs and temporal interactions between neurons in an AI column. Data are from the example penetration shown in Fig. 1. Each row represents a separate pair of neurons. (Left) Depth, firing rate, and STRF of the neurons for which cross-covariance functions were computed. Layer assignments are to the right of the STRFs (L2-L6). (Right) Cross-covariance functions for the pairs of neurons in the left column. Arrows indicate direction of the temporal interaction: negative delays mean A fired before B, positive delays mean B fired before A. Dashed lines indicate 99% confidence intervals. Gray curves indicate shift predictors, i.e., the timing distribution of non-simultaneously recorded spike trains. The laminar connection patterns most consistent which the cross-covariance function are shown to the right of the each STRF pair.

The goal of this study was to determine the relationship between spectrotemporal processing and functional connectivity in the AI microcircuit. To quantify connectivity, we computed cross-covariance functions between the spike trains of neurons in multi-channel probe penetrations. For the 15 neurons in the example penetration (Fig. 1), this resulted in 105 cross-covariance functions. Five examples are shown in Fig. 2. Each row represents one pair of neurons. The left column shows the STRFs of each pair of neurons. The right column displays the cross-covariance functions for each pair, along with the corresponding 99% confidence intervals (dashed lines). For these cross-covariance functions, peaks to the right of zero delay indicate that neuron B fired before neuron A. Since neural response correlation may be due to stimulus effects, we also calculated shift predictors, which were based on two presentations of the ripple stimulus (Fig. 2, gray curves). Shift predictors were always smaller in magnitude than cross-covariance functions. This indicated that the connectivity was likely due to neural connectivity and not simply a result of stimulus synchronization [53], [54]. Following previous arguments, it is unlikely that the auditory system performs a stimulus-induced correlation as estimated by the shift predictor, since the brain has access to only one stimulus instantiation [30], [54], [55], [56]. Thus, the actual spike coincidences affect the firing of the target neuron, not the stimulus-corrected ones, since the auditory cortex cannot calculate shift predictors [30], [57]. For this reason, we do not consider the shift predictor in further analyses.

The connectivity patterns for these example pairs were consistent with the known vertical feedforward and feedback circuitry of cat AI. The cross-covariance function in the first row shows that neuron A (650 µm) fired before neuron B (800 µm), as indicated by the delay at which the peak activity occurs, and by the function's center of mass. This pattern is consistent with a short layer 3 to layer 4 feedback connection. The second row shows a feedforward connection from layer 3 (650 µm) to layer 2 (350 µm), also consistent with AI connection patterns [7], [8], [14]. The third example shows a broader covariance function, which is consistent with a layer 5 (1100 µm) to layer 2/3 (350 µm) feedback connection. The increased width of the function may be due to the synaptic distance between layer 5 and layer 3, which allows other synaptic connections to cumulatively effect neural synchronization. The fourth example shows layer 3 spiking leading layer 5 responses, corresponding to a major feedforward branch of the microcircuit [8]. Finally, the last row shows a cross-covariance function that is consistent with a layer 6 to layer 4 connection, which is known to be present in AI [8], [58].

**Figure 3:**
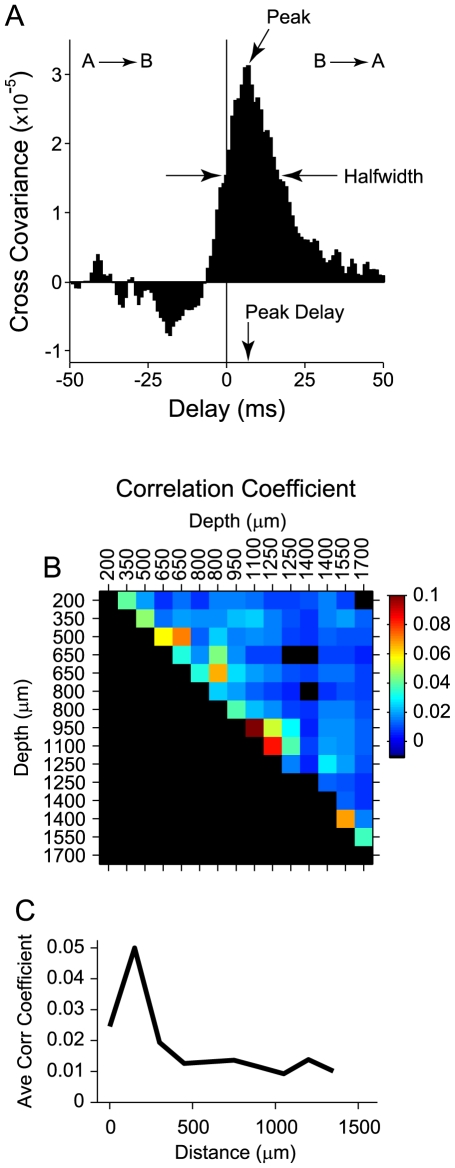
Analysis of cross-covariance functions. (A) Peak value, delay at which the peak occurs, and cross-covariance function halfwidth were extracted from each cross-covariance function. Peak delay and halfwidth are calculated with respect to the maximum (Peak) in the function. (B) Correlation coefficient depth matrix, calculated from cross-covariance functions for the data in Fig. 1. Since the matrix is symmetric, values below the diagonal are not shown. Black squares above the diagonal indicate non-significant connections. Duplicated depth values represent neurons recorded at the same depth. (C) Correlation coefficient versus distance between neurons for the data in (B).

To quantify neural synchronization within the cortical column, we extracted several parameters from the cross-covariance functions (Fig. 3A). Cross-covariance functions were only analyzed if at least two consecutive bins in the function exceeded the 99% confidence limits. Three parameters were extracted: (1) the peak correlation coefficient value; (2) the delay at which the peak response occurred; and (3) the halfwidth, which is the width of the cross-covariance function at half its peak value.

For the example penetration in Fig. 1, we examined the peak correlation coefficient for all possible pairs of neurons. We made comparisons by plotting the possible pair combinations in matrix form. The depth value of each neuron is indicated to the left and above each matrix. Repeated matrix depth values indicate different neurons recorded from the same electrode channel. The value of a matrix element equals the parameter value (Fig. 3B). The matrix element (350, 500), with the row value of 350, and the column value 500, represents the correlation coefficient for a pair of neurons, with one neuron at 350 µm and the other at 500 µm.

Peak correlation coefficient values, which reflect the functional connection strength between neurons, were greatest for nearby neurons (Fig. 3B for the example penetration of Fig. 1; black squares above the diagonal indicate non-significance; values below the diagonal are not shown since they are identical to those above the diagonal). Values decreased for increasing laminar and synaptic distance. By examining the distance between neurons, we found that for the example penetration the connection strength was strongest for neurons that were separated by the shortest distances (Fig. 3C). Consistent with the idea that strong neuronal coupling is present in granular layers, the strongest connections were observed at depths corresponding to layer 4 (950 and 1100 µm).

**Figure 4:**
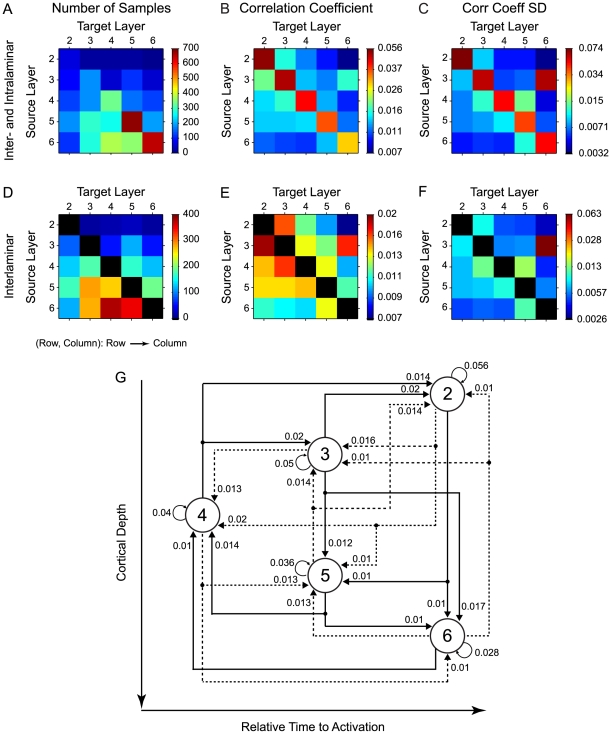
Summary of AI interlaminar connection strengths. For each neuron pair with a significant correlation we determined the direction of the connection and the correlation coefficient, or connection strength. (A) Number of pairs for all inter- and intralaminar data, grouped by laminar connection pattern. Each element in the matrix is in Row to Column form (Row, Column), which implies that the connection was from *Row* ( =  source layer) to *Column* ( =  target layer). The parameter value is indicated by the color. Element (4,3) implies the number of significant connections from layer 4 to layer 3 in our dataset. (B) Mean inter- and intralaminar connection strength, or correlation coefficient, for all significantly connected neuron pairs. (D, E) same as (A,B), with the intralaminar contributions removed to emphasize patterns of inter-laminar connections. (D) Number of connected pairs for each interlaminar combination. (E) Mean inter-laminar connection strength. (C, F) Standard deviation (SD) of inter- and intralaminar connection strength distributions, corresponding to the data in (B,E). (G) Layer connectivity diagram for the data in (B). Interlaminar and interlaminar connections are shown. Solid lines indicate feedforward connections in the auditory cortical microcircuit. Dashed lines indicate feedback connections. Values indicate the connection strengths from (B). Layers, indicated by circles, are vertically arranged to coincide with cortical depth. Layers are also organized horizontally to indicate the relative response time of each layer, as determined from latency analysis [37].

We further examined the correlation coefficients, or connectivity strengths, for each pair of neurons in our dataset. Each neuron in a pair was assigned to the appropriate laminae (Fig. 4), and the parameter values were compiled into laminar matrices. Each element of a laminar matrix is denoted in (row, column)-form. Rows in the matrix correspond to the position of the source neuron (based on the polarity of the peak delay in the cross-covariance function), and columns represent the position of the target neuron. For example, element (4,3) represents a connection consistent with layer 4 projecting to layer 3. Element (3,4) represents layer 3 projecting to layer 4, while element (3,3) represents connections between neurons within layer 3. The value, or color, in each matrix represents the mean magnitude of the parameter. For most matrix elements we had over one-hundred pairs, with the highest number of encountered pairs (∼25%) in layers 5, and 6 (Fig. 4A,D), most likely due to their relatively larger thicknesses, and the larger cell sizes in those layers, making it easier to isolate single neurons in those layers [59], [60].

The layer-specific population distribution of connection strength was largely consistent with the known wiring in cortical microcircuits ([8], see Discussion). The average connection strength within and across layers is shown for all neuron pairs in matrix-form in Fig. 4B and as a circuit diagram in Fig. 4G. The strongest connectivity was clearly observed for neuron pairs located within each layer (Fig. 4B). These intralaminar pairs consist mostly of neurons in close spatial proximity. The standard deviation of the connection strength distributions followed a similar trend, with the greatest variability for intralaminar connection strength corresponding to elements in the connection strength matrices that had the highest values (Fig. 4C,F). Thus, the variability pattern is most likely similar to the connection strength pattern because the increasing range of connection strengths makes it possible for the variability to increase.

To emphasize the pattern of interlaminar connectivity, both the number of recorded pairs (Fig. 4D) and the average connection strength (Fig. 4E), were replotted, with the intralaminar pairs omitted. These off-diagonal connectivity values were usually half the magnitude of the diagonal, intralaminar elements. The highest interlaminar connectivity strengths (Fig. 4D, red squares) were observed for feedforward connections from layer 3 to layer 2, layer 4 to layer 3, layer 3 to layer 6, and for the feedback connection between layer 2 and layer 3. Intermediate connectivity strength was found for feedforward transfer from layer 4 to 2, as well as feedback from layer 5 to layers 2, 3, and 4. The weakest feedforward connectivity was observed between layer 2 to layers 5 and 6, and both feedforward and feedback connections between layers 4 and 6. The main difference to traditional connectivity schemes for auditory cortex was the surprisingly strong connection from layer 3 to 6 combined with a significantly weaker connection from layer 3 to 5 (p<0.01; Rank Sum test). This may suggest that the thalamo-cortico-thalamic loop incorporates delayed, secondary cortical processing - outside layers 4 and 6 - over potentially faster but less processed feedback signals directly conveyed from layer 4 to layer 6.

### Peak Delay and Halfwidth

**Figure 5:**
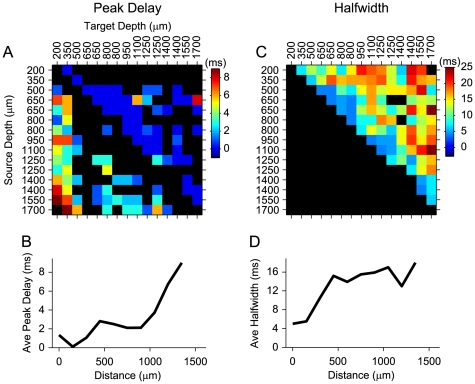
Example temporal interaction parameter matrices for neurons in an AI column. Data from example penetration in Fig. 1. Matrix values are indexed according to specific neuron combinations indicated by the depths listed above and to the left of the plots in (A, C). (A) Peak delays from cross-covariance functions. Absolute values of peak delays are shown. Each element in the matrix is in Row to Column form, (Row, Column), which implies that the connection was from *Row* to *Column*. The signs (+ or −) of peak delays were used to determine the direction of the connection. Black matrix elements indicate non-significant connections or connections not consistent with cross-covariance functions. (B) Average peak delay as a function of intra-columnar distance between neurons for the data in (A). (C) Halfwidths of cross-covariance functions. Values below the diagonal are not shown since the matrix is symmetric. Black matrix elements above the diagonal indicate non-significant connections. (D) Average halfwidth versus intra-columnar distance between neurons for the data in (C).

The delay of the peak in the cross-covariance function estimates the time elapsed between activity in the source and the target neuron. Delays increased as the physical distance between neurons increased (Fig. 5A for the example penetration of Fig. 1). The longest delays in this penetration were between sources in the infragranular layers and targets in layer 2 (Fig. 5A). The relationship between peak delay and distance between neurons for all pairs in this penetration (Fig. 5B) indicates that the largest distances correspond to the highest delay values. Halfwidth also increased with increasing distance between neurons (Fig. 5C,D). Across the penetration, the halfwidth of covariance functions achieved a minimal value of approximately 5 ms for separation distances of 0 µm, then increased rapidly for neurons separated by 0 to 500 µm, and then asymptoted at 16 ms from 500 µm to larger distances (Figure 5D).

**Figure 6:**
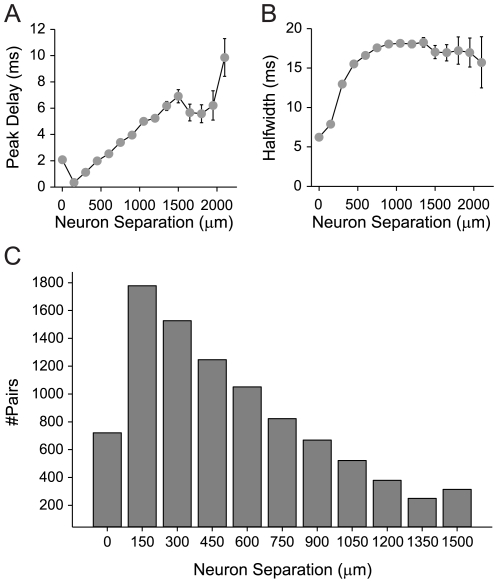
Temporal interaction parameters as a function distance between neurons. (A) Population data for peak delay versus neuron separation. Cross-covariance function peak delay increases with increasing cortical distance between functionally connected neurons. Data are mean +/− S.E.M. (B) Population data for halfwidth versus neuron separation. Halfwidth increases with neuron separation. Data are mean +/− S.E.M. (C) Frequency histogram of neuron separation for functionally connected neurons.

For the population data, the cross-covariance peak delays were significantly correlated with neuron separation (Fig. 6A: r = 0.442, p<0.01, t-test). The slope of the relationship between delay and distance indicates an average columnar propagation velocity of 0.22 m/s, which is very similar to a previous estimate of 0.26±0.05 m/s in a slice preparation [61]. The highest proportion of connected neurons was within 300 µm of each other, although many connected pairs were found at larger separation distances (Fig. 6C). Thus, the probability of a functional connection increases as the distance between neurons decreases. This is consistent with findings from in-vitro studies, which show that the probability of pyramidal cell communication is greatest at short separations [17], [62]. These findings suggest temporal response influences due to conduction distance and/or synaptic distance. Similar to the connection strength, it reflects an ordinal and orderly temporal sequence of processing within the columnar circuit.

The halfwidth of the covariance functions reflects various aspects of the response relationship between neurons including synchrony, intrinsic temporal response patterns, such as bursting or oscillatory events, and the influence of potential multiple inputs common to both neurons. As indicated by the example penetration (Fig. 5C,D), the halfwidth varied with cortical distance between the locations of the neurons. The narrowest functions arose from nearby neurons, especially in the thalamo-recipient layers. This is consistent with strong coupling between neurons in granular layers, a less variable cell population in granular layers, and the accumulation of synaptic jitter and the influence of multiple neurons as the distance between neurons increases.

The halfwidths of cross-covariance functions were also significantly correlated with neuron separation (Fig. 6B: r = 0.426, p<0.01, t-test). Neurons located within 500 µm of each other in a column showed the largest halfwidth change with separation, at a rate of ∼2 ms/100 µm. Beyond 500 µm separation the cross-covariance halfwidth remained fairly constant at ∼17 ms. The separation dependence over shorter distances likely reflects synaptic accumulation, where increasing distance between neurons allows synapses of other neurons to have a greater effect on synchronization or to provide common inputs, resulting in an increased range of the spike timing between cortical pairs (Figure 6B). The constant width for larger separations may be indicative of constraints on the effective columnar integration time. The width of correlation functions for horizontal connections in cat AI with matched characteristic frequencies have been shown to overlap with the distribution of halfwidths here [29]. In a previous study [25], however, correlation halfwidths of pairs isolated from the same electrode, i.e., most likely residing in the same lamina, were significantly broader than those found here (mean halfwidth 27 ms). Correlation functions between different columns were found to be even broader (mean halfwidth 42 ms; [25]).

### Synchronization across Layers

**Figure 7:**
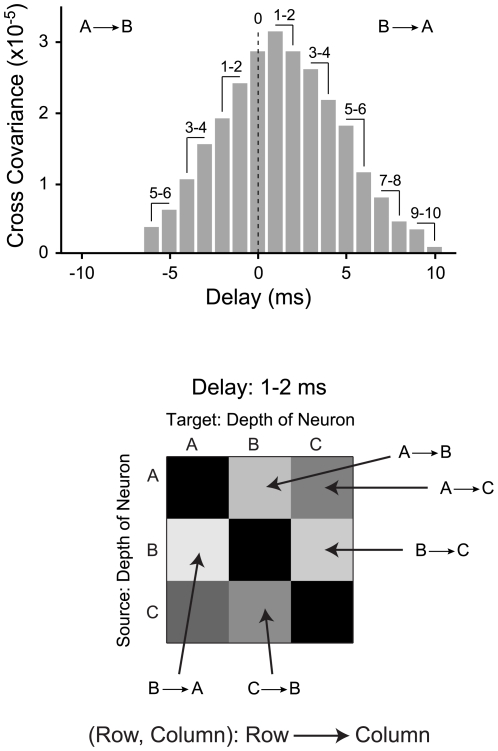
Method for obtaining values for Synchronization Matrices (SMs). (Top) Example cross-covariance function. Values for SMs are obtained by averaging cross-covariance function values at different spiking delays (1 and 2, 3 and 4, 5 and 6, 7 and 8, and 9 and 10 ms delays). The same procedure is used for negative delays. Only significant cross-covariance function values are shown. Non-significant values (−7 to −10) are excluded. (Bottom) Example SM for a delay of 1–2 ms for 3 neurons in a penetration. Each element in the SM represents a connection strength and connection direction. Elements are ordered so element (Row, Column) in the matrix indicates cross-covariance function values consistent with a Row (source layer) to Column (target layer) connection.

The temporal evolution of neural synchronization across laminae can provide important insights into the structure of the local circuit. For the example penetration (Fig. 1), we examined all cross-covariance functions, and extracted the values for different spike time delays. We analyzed eleven delay bins between −10 ms to 10 ms, using only covariance values that exceeded the 99% confidence intervals. The values were obtained at positive and negative delays, and statistically significant covariance function values were averaged for delays of 0 and +/− 1–2, 3–4, 5–6, 7–8, and 9–10 ms (Fig. 7, top, shows an example of this procedure).

**Figure 8:**
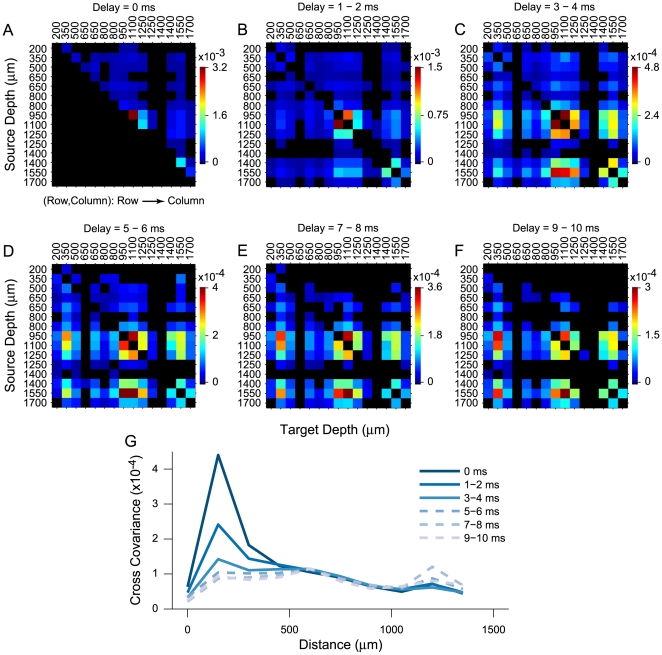
Synchronization Matrices (SMs) for neurons in an AI column. Data from example penetration in Figure 1. (A–F) SMs. Each pixel in an SM represents the strength of the cross-covariance function between the neurons whose positions are listed above and to the left of the plot (blue to red indicates increasing connection strength). Matrices are ordered so that element (A,B), or (Row, Column), represents the cross-covariance function value consistent with an A to B flow of information. The SM values are obtained by averaging the cross-covariance function values at the delays listed above each plot. The strength of neural synchronization between local neurons decreases for longer delays but stays the same or slightly increases between more distant neuron pairs (increasing SM values at off-diagonal positions). Black pixels indicate cross-covariance function values that did not achieve significance. (G) Cross-covariance function values versus distance between neurons at multiple delays. Data are obtained from the SMs in (A–F).

The averaged covariance function values were placed in Synchronization Matrices (SMs; Fig. 7, bottom). The SMs show neural coordination for a single probe penetration, and summarize the temporal change in functional connectivity recorded by the entire multi-channel electrode. SMs for the data in Fig. 1 are shown in Fig. 8A–F. Each matrix displays significant covariance function values at different delays (non significant values are shown in black). The position of each neuron in the column is shown to the left and on top of each SM. An SM is organized so that a given element in the matrix relates to a covariance value at a specified delay. Each matrix element, given in (Row or A, Column or B) notation, is interpreted as a covariance function value for a projection from the neuron at depth A to the neuron at depth B. The example in Fig. 8C shows that at delays of 3–4 ms, there is joint activity between neurons at 1100 µm and 350 µm, indicating that there was significant correlated activity between cells in layer 5 and layer 3. Since the element is (1100, 350), the information is directed from source layer 5 to target layer 3. In another example, there is correlated activity between neurons at 1550 µm and 800–1100 µm, corresponding to information directed from layer 6 to layer 4. Finally, coordinated activity near the diagonal, for neurons located at 800–1250 µm, indicates reciprocal short-range neural coordination. Inspection of the SMs for different delays reveals that coordinated activity begins with synchronous responses at 0 ms delay, mostly between neurons located in layers 4 and 5. At 3–4 ms delays, coordinated and reciprocal activity spreads and emerges between neurons at 950–1250 µm and 1400–1550 µm (layer 4, 5, and 6). Coordinated activity is also present between neurons at 1400 to 1700 µm, corresponding to layers 5 and layer 6. At longer delays, responses of neurons at 950, 1100, and 1550 µm leads to responses of a neuron at 350 µm. At delays greater than 3–4 ms, increasing numbers of elements in the SMs indicate non-significant interactions. This decrease in significant interactions reflects the duration of the covariance function widths. Thus, most significant coordinated activity is present for approximately 10 ms, then steeply decreases. The co-variation strength versus distance profiles (Fig. 8G) summarize the temporal evolution behavior in a cortical column. The highest cross-covariance values were observed for short delays and for small separations indicative of local interactions. For longer delays, the cross-covariance values were reduced and much less dependent on neural separation. This indicates that the width of the cross-covariance function reflects coordinated inputs arising from different processing nodes in the columnar circuit, and the strength is dominated by local neurons less than 500 µm apart.

### STRF Similarity between Functionally Connected Neurons

STRF similarity is a parameter that may govern the probability of a significant connection. We explored this by comparing the structure of STRFs of functionally connected neurons. The similarity index is the correlation coefficient between two STRFs, and ranges from +1 for identical STRFs to −1 for STRFs that are anti-correlated [40]. For all significantly connected pairs, we computed three similarity indices: for the full STRF; for the excitatory subfields of the STRF; and for the inhibitory subfields of the STRF. We then compared the similarity index values to the correlation coefficient for each pair of neurons.

**Figure 9:**
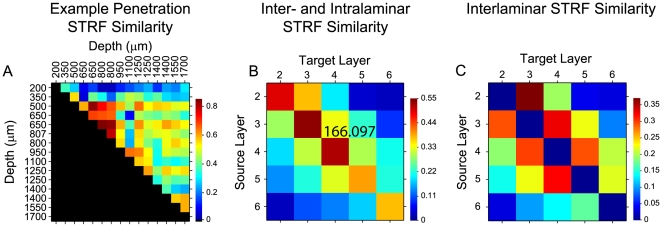
STRF similarity for functionally connected neurons. (A) Example STRF similarity index matrix for the neurons in Fig. 1. Each matrix element represents the similarity between the STRFs of different neurons. The similarity between STRFs is greatest between neurons at supragranular (200–800 µm) and granular (800–1100 µm) layer depths. Data below the diagonal are not shown since the matrix is symmetric. (B) Intra- and interlaminar STRF similarity across all data, grouped according to layer. STRFs are most similar for connected neurons within the same layer. (C) Interlaminar STRF similarity data (data from (B) with intralaminar data removed).

Most neurons in a given columnar penetration had correlated receptive fields (Fig. 9A for the example penetration of Fig. 1; values below the diagonal are not shown since the matrix is symmetric). Highly similar receptive fields clustered in depths corresponding to the thalamorecipient layers 3 and 4. STRFs in infragranular layers were less uniform, and were less similar to each other. Thus, though neurons in AI columns may share similar CFs (Fig. 1B), their receptive field structure need not be highly similar.

When we pooled the STRF similarity data into laminar matrices we uncovered two basic rules (Fig. 9B,C). First, the highest similarity of connected neurons was found within the same layer (Fig. 9B). Second, the similarity between STRFs increased as the distance between neurons decreased (Fig. 9C). When we compared STRFs, those between neurons in layer 4 and 3, layer 3 and 2, and layer 4 and 5 were the most similar. Pairs located in neighboring laminae were more similar than pairs in layers that were further apart. The lowest STRF similarity was seen between layers 2 and 6. We did not observe clear differences in similarity between feedforward and feedback pairs (A,B and B,A elements in the matrix of Fig. 9C).

**Figure 10:**
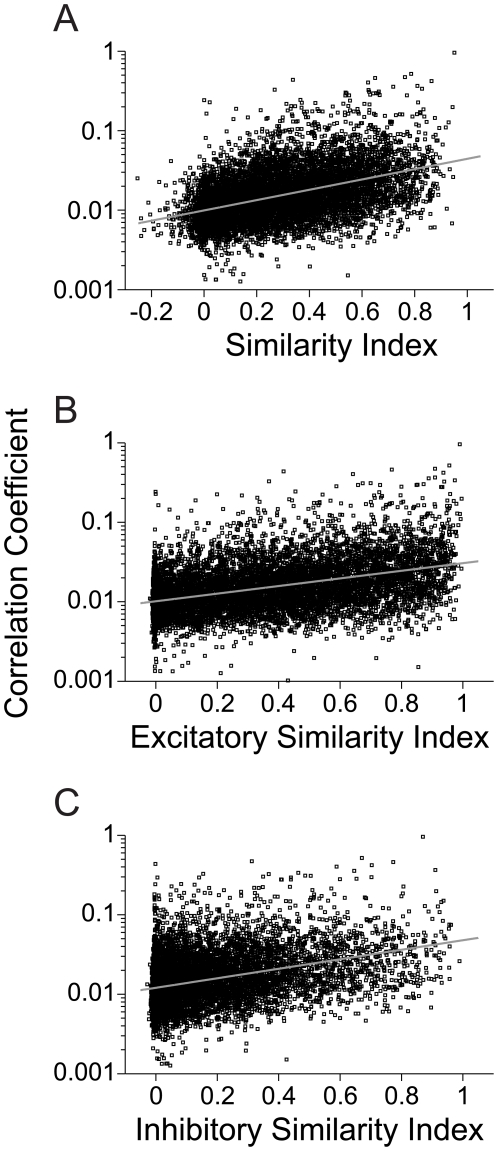
Connection strength versus STRF similarity. Correlation coefficient values as a function of (A) Full STRF similarity between functionally connected neurons (r = 0.464, p<0.01, t-test). (B) Similarity between only the excitatory STRF subfields (r = 0.433, p<0.01, t-test). (C) Only the inhibitory STRF subfields (r = 0.379, p<0.01, t-test). (N = 8364).

Next, we examined how functional connectivity relates to receptive field similarity. In our sample of significantly connected neurons, the correlation coefficient significantly covaried with the similarity index for the full STRFs (Figure 10A: r = 0.464, p<0.01, N = 8364, t-test). This correlation was higher than that for either the excitatory (Figure 10B: r = 0.433, p<0.01, t-test) or inhibitory (Figure 10C: r = 0.379, p<0.01, t-test) STRF subfields. Even though the excitatory subfield is more stereotyped and less variable along the column, the full STRF similarity, including excitatory and inhibitory subfields, appears to be a better predictor of connection strength.

The relatively modest correlation between connection strength and STRF similarity suggest that columnar wiring between AI neurons accounts for only a small portion of the evolving receptive field characteristics of the constituent neurons.

### Estimates of Monosynaptic Connections

**Figure 11:**
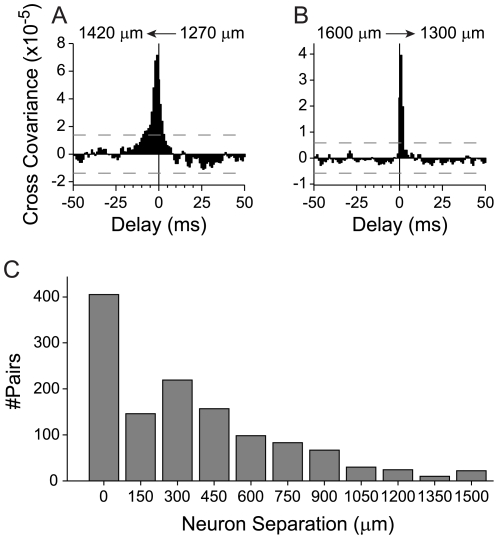
Putative monosynaptically connected neurons. Connections were classified as monosynaptic if peak delays were 1–4 ms, and if cross-covariance function halfwidths were less than 10 ms. (A,B) Examples cross-covariance functions for two putative monosynaptically connected neurons. (A) Functional connection between two cells in layer 5. The direction of the connection is from the cell at 1270 µm to the cell at 1420 µm. (B) Functional connection from a cell in layer 6 (1600 µm) to a cell in layer 5 (1300 µm). (C) Vertical distance between neurons for all monosynaptic connections (N = 1203 pairs).

To this point, we have not distinguished between polysynaptically or monosynaptically connected neurons. To distinguish these connections, we parsed our database of cross-covariance functions, and extracted functions that had peak delays between 1 and 4 ms, and halfwidths less than 10 ms (Fig. 11A,B: example cross-covariance functions). We assumed that neuron pairs satisfying these two conditions were more likely to be monosynaptically connected. The 10 ms halfwidth was chosen because monosynaptic connections are most likely to occur for neurons within 500 µm of each other [62], and 10 ms marks the boundary of our halfwidth population data between neurons separated by <500 µm (see Fig. 9B). The highest proportion of putative monosynaptically connected neurons was within 500 µm of each other (∼75%). At greater distances, the probability of a significant connection decreased exponentially (Fig. 11C). Despite this decrease, putatively monosynaptically connected neurons were found at separations >900 µm and may reflect the systematic vertical arrangement of neuron processes in AI [63]. The total proportion of narrow cross-covariance functions, or putative monosynaptic connections, was ∼17%, which corresponds closely to the proportion of 16% of unilateral excitatory inputs previously reported [25].

**Figure 12:**
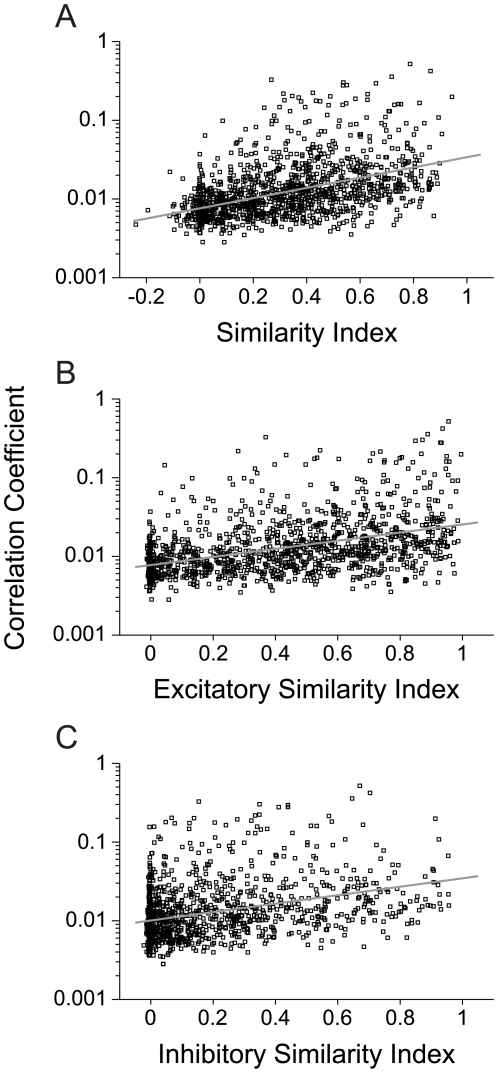
Correlation coefficient versus STRF similarity for monosynaptic functionally connected neurons (N = 1203 pairs). STRF similarity was computed for (A) the full STRF (r = 0.444, p<0.01, t-test), (B) the excitatory subfields of the STRF (r = 0.434, p<0.01, t-test), and (C) the inhibitory subfields of the STRF (r = 0.348, p<0.01, t-test).

STRF similarity covaried with correlation strength for the putative monosynaptic connections. The similarity indices for the full STRF were significantly correlated with correlation strength (Figure 12A: r = 0.444, p<0.01, N = 1203, t-test). This correlation was higher than that for the excitatory or inhibitory STRF subfields alone (Excitatory – Fig. 12B: r = 0.434, p<0.01; Inhibitory – Fig. 12C: r = 0.348, p<0.01, t-test). The correlation between connectivity strength and STRF similarity for the putative monosynaptically connected neuron pairs was not significantly different than that for the whole population (Fig. 10).

The similarity index is the correlation between the STRFs of two neurons. Thus, it correlates the spectrotemporal structure to which both neurons respond. Neurons that similarly respond to spectrotemporal stimuli may also share other STRF properties. Therefore, we pursued the question of how specific STRF metrics are transformed between neurons that are putatively monosynaptically connected. Thus, we calculated six additional STRF metrics, and examined how these metrics varied between functionally connected neurons. For each significant connection, we plotted the same STRF measure for the target (*Post*) neuron versus the measure for the source (*Pre*) neuron, as determined by the sign of the peak delay of the cross-covariance function (e.g., Fig. 2.).

**Figure 13:**
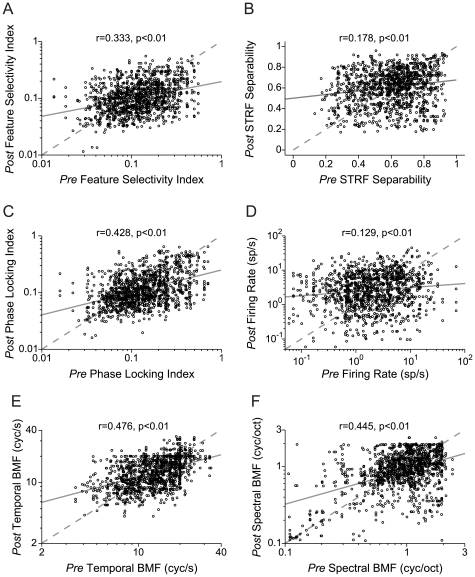
Comparison of receptive field parameters for monosynaptic functionally connected neurons (N = 1203 pairs). Each data point represents a connected pair. The abscissa (*Pre*) represents the parameter value for the neuron in the pair that responded first, or was *Presynaptic*, according to the peak delay in the cross-covariance function. The ordinate (*Post*) represents the neuron whose response came after the other neuron in the pair, or was *Postsynaptic*. (A) Feature Selectivity Index (r = 0.333, p<0.01). (B) STRF Separability (r = 0.178, p<0.01). (C) Phase Locking Index (r = 0.428, p<0.01). (D) Firing Rate (r = 0.129, p<0.01). (E) Temporal Best Modulation Frequency (r = 0.476, p<0.01). (F) Spectral Best Modulation Frequency (r = 0.445, p<0.01; t-test used for all comparisons).

Figure 13A displays the feature selectivity index (FSI) values for each member of a functionally connected pair. The FSI reflects the stimulus selectivity of a neuron, and quantifies the degree to which the stimulus segments that were used to construct the STRF match the STRF. The FSI values of the source and target neurons are only moderately correlated (r = 0.333, p<0.01, N = 1203, t-test) between connected neurons. Furthermore, the difference between the FSIs of the two cells is weakly correlated with the connectivity strength (r = −0.177, p<0.001, N = 1203, t-test). Neurons with similar FSIs are more likely to be functionally connected, but the degree of feature selectivity is subject to substantial modification by target neuron processing.

Next, we examined the structure of the STRFs of putative monosynaptically connected neurons. For each STRF, we calculated the separability index, and thus determined the degree to which the STRF can be decomposed into a product of two one-dimensional, independent functions of spectral and temporal processing, respectively. If it is highly separable, then time and frequency are dissociated in the STRF. An index of 1 indicates complete dissociation, while a value of 0 indicates the opposite. The separability indices (Figure 13B: r = 0.178, p<0.01, t-test) of monosynaptically connected neurons were also weakly correlated. The correlation between the difference of the separability indices and the connectivity strength was again low (r = −0.045, p<0.01, t-test) indicating that functional connectivity is not a good predictor of spectrotemporal interactions.

Monosynaptically connected pairs similarly phase locked to the spectrotemporal structure of the stimulus envelope (Figure 13C: r = 0.428, p<0.01, t-test). The difference in phase-locking ability between the two neurons showed the strongest correlation with connectivity strength of the STRF parameters, though it was still only moderate (r = −0.287, p<0.01, t-test). This indicates that these connected pairs have similar temporal precision when they respond to acoustic stimuli. By contrast, evoked firing rates were only weakly correlated with monosynaptic connectivity (Figure 13D: r = 0.129, p<0.01, t-test).

Spectral and temporal modulation properties showed a relatively high congruence between more strongly connected neurons. Best modulation frequencies were well correlated for monosynaptically connected neurons. Best temporal modulation frequency had the highest predictive value (Figure 13E: r = 0.476, p<0.01, t-test), while best spectral modulation frequency was slightly less correlated (Figure 13F: r = 0.445, p<0.01, t-test). Thus, the best predictors of functional connectivity from receptive field properties were phase locking, best temporal modulation frequency, and best spectral modulation frequency.

Following earlier reports, we also analyzed how halfwidth varied with receptive field parameters [29]. Halfwidth may be an important variable, it is relatively uncorrelated with correlation strength, and since it varies with pure-tone receptive field properties, as opposed to correlation strength, which was found to vary with the response properties of neurons [29]. We also found a weak correlation between connection strength and halfwidth (r = −0.152, p<0.01), and thus we investigated the correlation between halfwidth and other STRF parameters. Over the monosynaptic data, halfwidth was significantly correlated with STRF similarity (r = −0.322, p<0.01), the difference in FSI (r = −0.094, p<0.01), and the difference in best spectral modulation frequency (r = 0.0743, p = 0.01). Thus, as the STRFs of connected cells become more similar, the width of the correlation function decreases. It follows that narrower halfwidths correlated with more similar best spectral modulation frequencies. This may indicate that nearby neurons share modulation properties, since smaller halfwidths are more likely as the separation between neurons decreases (Fig. 6). Less interpretable is the decrease in FSI difference with increasing halfwidth. The correlation in this case, however, was less than 0.1, indicating that it is a weak, and ambiguous, indicator for connectivity relationships.

## Discussion

Our goal in this study was to dissect the functional connectivity between neurons in the vertical or columnar AI microcircuit. We took advantage of two methodological approaches. First, multi-channel probes allowed us to simultaneously sample from single neurons in each layer of AI. Second, we coupled this with the presentation of a dynamic moving ripple stimulus, and then constructed the STRF of each neuron. This integrated approach allowed us to quantify the functional connectivity between neurons in different layers, and then relate this to receptive field properties of each neuron.

Our approach had several key advantages. First, by using the STRF as an assay for auditory processing we were able to capture the major mode of auditory cortical processing. The STRF represents the dominant acoustic features to which a neuron responds. By analyzing it, we quantify the spectrotemporal features that best predict a neuron's stimulus preferences. Second, by using cross-covariance functions we were able to quantify functional connectivity in a rigorous, parametric manner. Background activity is removed from cross-covariance functions, and thus the functions reflect the actual functional influences of one neuron on another without the confounding factor of background coincident spiking. Using the cross-covariance function also allowed us to place rigorous statistical bounds on our estimates. Third, by only analyzing cross-covariance functions with two consecutive significant bins, we drastically reduced the chance that spurious false-positives affected our results. This is because the false positive rate is one out of one-hundred at our significance level of 0.01. Further, requiring two consecutive bins to reach the 0.01 level makes the influence of spurious coincidences even less likely. Fourth, the previous considerations make the use of the correlation coefficient and halfwidth metrics even more compelling. The correlation coefficient was calculated with respect to the peak in the correlation function. Since we required two consecutive significant bins, it is unlikely that this metric is biased by random fluctuations. Also, since we removed background activity, the halfwidth represents an estimate of the driven temporal overlap for the responsiveness of pairs of neurons. Thus, the halfwidth metric is not an overestimate of the duration of temporal interactions.

Along with these strengths, a few general observations and caveats should be mentioned. With regard to spike-sorting, single neurons were isolated using a Bayesian spike-sorting algorithm [34] that allowed the identification and separation of waveforms recorded on the same channel, even in the case of overlapping waveforms. Consequently, full correlograms could be reconstructed for neurons recorded on the same electrode for short delays. Second, our analysis focused solely on excitatory connections. While some putative inhibitory interneurons have been identified [50], we did not see unambiguous signs of inhibitory troughs in the cross-covariance functions. This is in agreement with previous correlation studies (e.g., Eggermont, 1992), however, the cause for this potential bias or incompleteness remains unclear. A more focused experimental approach, with the recording electrode tailored to the desired neuron type [64], may be required to obtain evidence of inhibitory temporal interactions [65]. Third, our analysis was based on cross-covariance functions, and in every case the accompanying shift predictors were smaller than the estimated functions. While this likely ensures that connectivity driven influences are the main determinants of our results, other subtle remaining effects cannot be ruled out.

Our study is the first in auditory cortex to focus on functional connectivity within the entire vertical microcircuit. Studies of horizontal connectivity are more numerous, and share some broad similarities with our results. Functional connectivity increases for horizontal connection when receptive field properties are similar. This holds for pure-tone receptive fields [29] and for STRFs [56]. Additionally, using noise-like stimuli reduced background activity and correlations [56]. The ripple stimulus we used is particularly appropriate in this regard, since it reduces the background oscillations that can confound connectivity studies [66]. The effects of the reduction in background activity can be seen in the example shift-predictors, which were always reduced in magnitude relative to the analyzed connectivity functions.

Several results from horizontal studies differ from our findings. Most basically, the anatomical connectivity scheme within columns is fundamentally different from that for horizontal connections [8], [14]. Additionally, for recordings from two electrodes in an AI iso-frequency contour, the halfwidths of cross-correlation functions are larger than those in our report [29]. They ranged from 10 to 100 ms, which is much greater than the mean halfwidths of approximately 17 ms that we obtained in the columnar circuit (Fig. 6B). Part of this difference may be due to the greater chance to accumulate synaptic jitter over longer connections distances. The results of the iso-frequency study [29] may also differ from ours because of the nature of the recordings in that report (multi-unit), the pre-processing of the data (smoothing of correlation functions), the definition of the halfwidth used (which did not exclude background activity), and the larger CF discrepancy of the pair's (mean difference was 0.45+/−0.42 octaves). By comparison, the average CF disparity between neurons within each of our vertical penetrations was only 0.1+/−0.1 octaves.

For horizontal connections, single unit analysis has revealed that the halfwidths of correlation functions are smaller than those for multi-units [25]. In the case of multiple single units, mean halfwidths ranged between 27 and 42 ms, indicative of greater overlap in the time of response for simultaneously recorded units compared to our data for vertical connectivity. An interesting finding in the single-unit horizontal connectivity analysis was that the connectivity between nearby units decreased as the depth of the recorded neurons increased [25]. This parallels our results, which revealed that same layer connection was greatest in supragranular layers and lower in infragranular layers (Fig. 4). A hypothesized reason is that the decrease in connection strength is due to decreasing common input with depth [25]. Another possible reason for the greater strength of supragranular connections is the greater synaptic efficacy between neurons within layer 2/3, within layer 4, and between neurons in layer 4 and layer 2/3 [67], [68], [69]. In these layers, synaptic efficacy is very strong, which leads to greater synchronization and connection strengths. These results were obtained from barrel cortex, however, and thus their analogy to auditory cortex remains a possibility that needs to be addressed for this discussion to rise above the level of speculation [25].

One final consideration concerns the use of the cross-covariance as a metric for quantifying functional connectivity. Since for each penetration we recorded multiple neurons, restricting our analysis to pairs of neurons may have overlooked interactions involving more than two neurons. Alternatively, if pair-wise interactions account for the majority of the total information in a network, then it is appropriate to restrict connectivity analyses to correlation or covariance functions for pairs of neurons [70]. To test this possibility requires that the information from pair-wise interactions be compared to the total information from all possible interactions. Such analyses have been completed in retinal and cortical slices. For retina, pair-wise interactions account for 90–99% of the total information available in the spiking responses of a population of neurons [71], [72]. In cortical slices, pair-wise interactions between neurons in different laminae account for 88% of the total information [70]. Thus, by using covariance functions, it is likely that we can quantify the majority of functional connectivity in the auditory cortical column.

### Temporal Sequence of Functional Connectivity

To study the temporal flow of acoustic information in the vertical AI microcircuit, we examined the cross-covariance functions between neurons located at different depths. The temporal interactions between neurons resembled the basic circuit diagram of laminar connectivity that was derived in earlier studies [7], [8]. Cross-covariance peaks occurred at delays which indicated that information was transferred from layer 4 to supragranular layers 2/3, then from layer 2/3 to layer infragranular layers. This pattern has been found in other sensory cortices [22], [73], [74] and may be a reflection of a uniformity of cortical architectonic organization [75], and of cortical modularity [76], [77]. We also found significant connectivity from layer 3 to layer 2, and from layer 5 to layer 3. These patterns have been described in-vitro, though they are sparsely seen in the intact animal [16], [22]. An unexpected observation was that the connectivity from layer 3 to layer 6 was significantly stronger than from layer 3 to 5. We conclude that the basic columnar microcircuit in AI is in general agreement with that in visual and barrel cortex [31], [74], though some differences in individual connectivity values between layers may prove significant for the interpretation of columnar transformations and information streams. It follows that dissecting the connection patterns in AI may shed light on those of other cortical regions. Additionally, these connection patterns may allow the cortex to perform similar computations in different sensory areas. This may be a consequence of the general excitatory circuit that is present in neocortex [74]. The representation of specific receptive field parameters, however, will vary between sensory modalities. Different representations are expected, since each system has connection patterns and neural response properties that are unique. The more pronounced connection of layer 3 to layer 6 than from layer 4 to layer 6 may indicate that cortico-thalamic feedback requires more elaborate preprocessing than in other modalities. Other examples of cortical properties unique to AI include callosal projections over the majority of the tonotopic map [78], the preponderance of pyramidal cells in layer 4 [79], and the fast kinetics of AI cells [80].

### Influence of Functional Connectivity on Receptive Fields

A significant result from our study was that the relation between receptive field structure and functional connectivity systematically varies with cortical depth and/or laminar position. Functionally connected supragranular neurons have similar receptive fields, though connected infragranular neurons do not share functional properties to the same degree. This implies that different rules, and thus functions, govern the connectivity of neurons in each layer. Neurons in layers 2 and 3 are the main contributors to corticocortical connectivity [81], [82]. Layers 5 and 6 provide large-scale projections to subcortical areas, even reaching down to the cochlear nucleus [83], [84]. These differences suggest that supra- and infragranular neurons may combine inputs in different ways, leading to varied, and likely layer-specific receptive field structure between connected neurons. This conclusion is supported by recent work showing that neurons in lower layer 3 and layer 4 have - nonlinearities that are structurally different from those in infragranular layers [37]. The nonlinearities of thalamorecipient layer neurons are consistent with STRFs that process stimuli more independently than the STRFs of neurons in deeper layers. Additionally, functionally connected infragranular neurons may have different STRF structure because they are selective for a wider range of stimulus features. This would lead to a decrease in STRF similarity between infragranular neurons since these neurons may respond to more than one stimulus dimension [85]. Thus, the stimulus preferences of these neurons cannot be completely captured by the spike-triggered averaging methodology employed in our report.

Does receptive field similarity predict functional connectivity? An affirmative answer implies that as the spectrotemporal preferences of neurons converge, their connection strength will increase. Our results do not support this claim. We found that functional connectivity was moderately predicted by receptive field similarity (Fig. 10, 12). This connectivity may also be explained by the position of neurons within the AI network. As the CF between neurons becomes similar, the inter-laminar connectivity increases. Thus, the main determinant of connectivity is the mapping of the sensory epithelium onto the surface of AI. Between layers, connectivity was not strongly predicted by STRF similarity (Fig. 10, 12), with the highest inter-laminar connection strength reaching only 0.1 (Fig. 4), though across the population data the highest strength was approximately 0.05 (Fig. 4). This implies that a minimum of 10–20 neurons projects to, and influences, the output of a postsynaptic cell.

An alternative explanation may be that we did not appropriately control for the manner of connectivity, and were not strict enough in examining only strongly monosynaptic connections (though see Figures 11 and 12). However, results from simultaneous recordings across the thalamocortical synapse are consistent with the results in this report [86]. When the CFs of a thalamic and a layer 4 cell are within 0.05 octaves, the monosynaptic connection probability is 30%. Despite this relatively high probability, there is no correlation between the strength of connection and CF difference. Additionally, receptive field similarity between connected thalamic and AI neurons is uncorrelated with connection strength [86]. This implies that across the thalamocortical synapse, where connections are expected to show less diversity, receptive field similarity is an inadequate predictor of connectivity. Thus, beyond CF, a definitive receptive field predictor of cortical connectivity is still unknown.

### Temporal Interactions in Other Sensory Cortices

Our results are broadly consistent with those in visual and barrel cortex. The basic circuit describing connections between layers appears similar across sensory modalities. Thus, visual and barrel cortex also follow the layer 4, layer 2/3, layer 5, layer 6, and layer 4 sequence of information processing [20], [21], [74]. These temporal interaction patterns are predicted by anatomical work in each cortex. In primary visual cortex, studies have shown direct connections from layer 4 simple cells to layer 2/3 complex cells [19]. This is consistent with the hypothesized feedforward model, where layer 4 simple cells project to neurons in layer 2/3, thereby creating classes of complex cells [87]. Though auditory cortex does not contain similarly qualitatively distinct functional cell classes, our work explicitly integrates receptive field parameters and functional connectivity. In sensory modalities, the basic excitatory connection patterns between layers are similar. We thus predict that receptive field processing will be a principle arising from connection patterns. In this context, processing is related to the dimensionality of the receptive field that is needed to adequately describe a neuron. From earlier work, we predict that neurons in layer 4, which can be described adequately by single STRFs, will project onto layer 2/3 neurons, thus leading to the extended receptive field model needed to describe these neurons [37]. Thus, while the receptive field properties differ, the laminar organization of receptive field processing will be similar, although not necessarily identical, to that in primary visual cortex, as indicated by some variations in the connections between supra- and infragranular layers in AI. Further studies that are focused explicitly on the connectivity and functional transformation between AI layers are still required [19].

### Conclusion

The basic connection patterns between AI layers, as demonstrated in anatomical studies, can be observed in functional connectivity studies using stimuli that are appropriate for spike-triggered analysis approaches. This implies that general neural circuit questions in AI can be addressed profitably with current stimulation and recording approaches. Our work shows that connectivity is largely similar in different sensory systems of the cat cortex with some variation in the connection strength between supra- and infragranular layers. We find that connectivity relates to receptive field structure differentially in various layers, since connected supragranular neurons had receptive fields that were more similar than the receptive fields of connected infragranular pairs. For putative monosynaptic interactions, temporal precision, feature selectivity, and modulation processing were moderately similar for functionally connected pairs. A strong functional predictor of connectivity was not found, likely due to the heterogeneous nature of AI receptive fields in different laminae. Taken together, these results constrain the response characteristics that are shared between, and perhaps govern, functional connections in AI layers and columns.
